# Supplementary material for: Skeletal muscle mitochondrial volume and myozenin-1 protein differences exist between high versus low anabolic responders to resistance training
Source: PeerJ. 2018 Jul 27;6:e5338. doi: 10.7717/peerj.5338 (PMC6065464; doi:10.7717/peerj.5338)
Supplement: Supplemental Information 2 [file peerj-06-5338-s002.zip › Raw western images, supplemental file 2.pptx]

## Slide 1
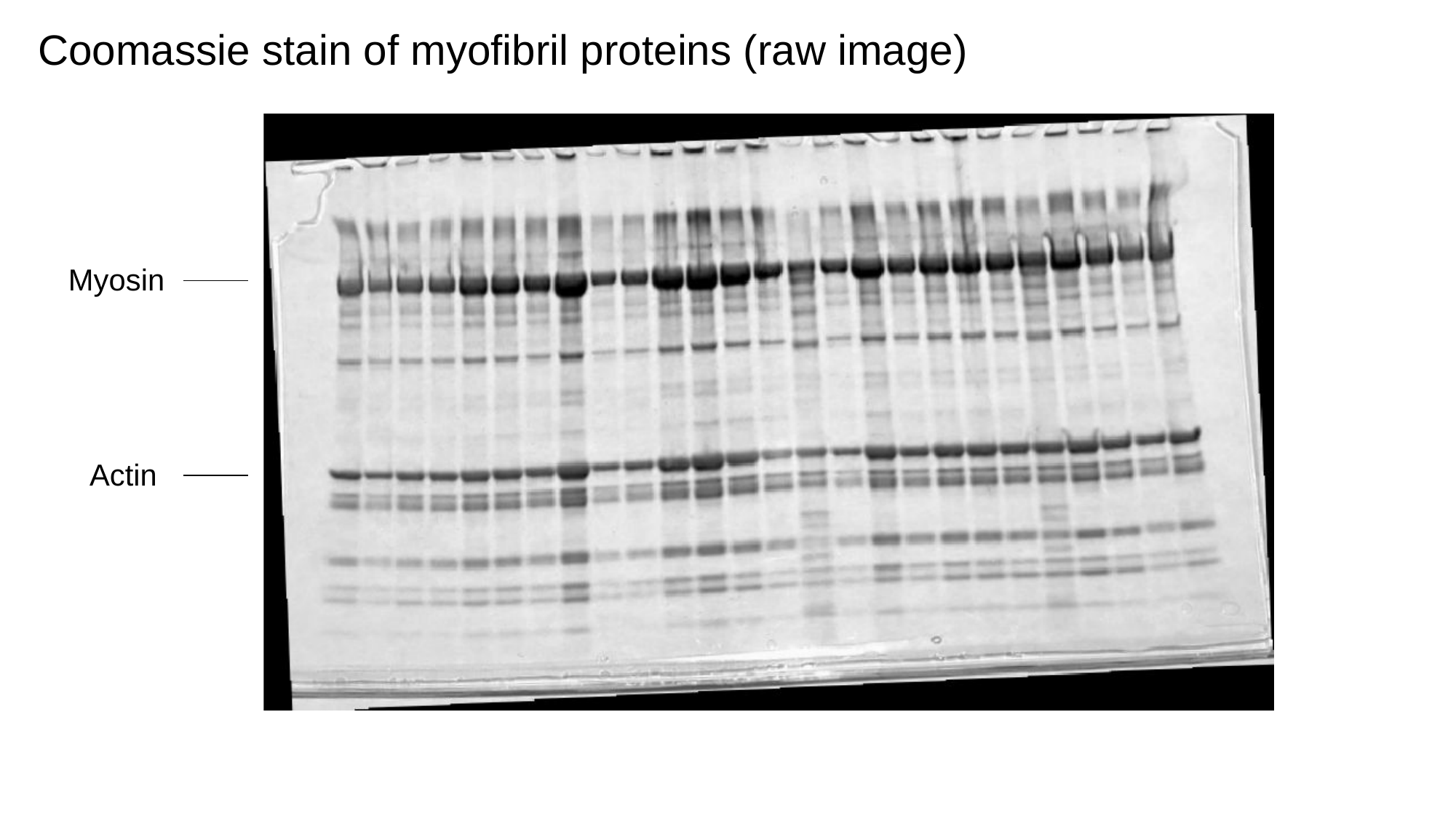

Coomassie stain of myofibril proteins (raw image)
Myosin
Actin

## Slide 2
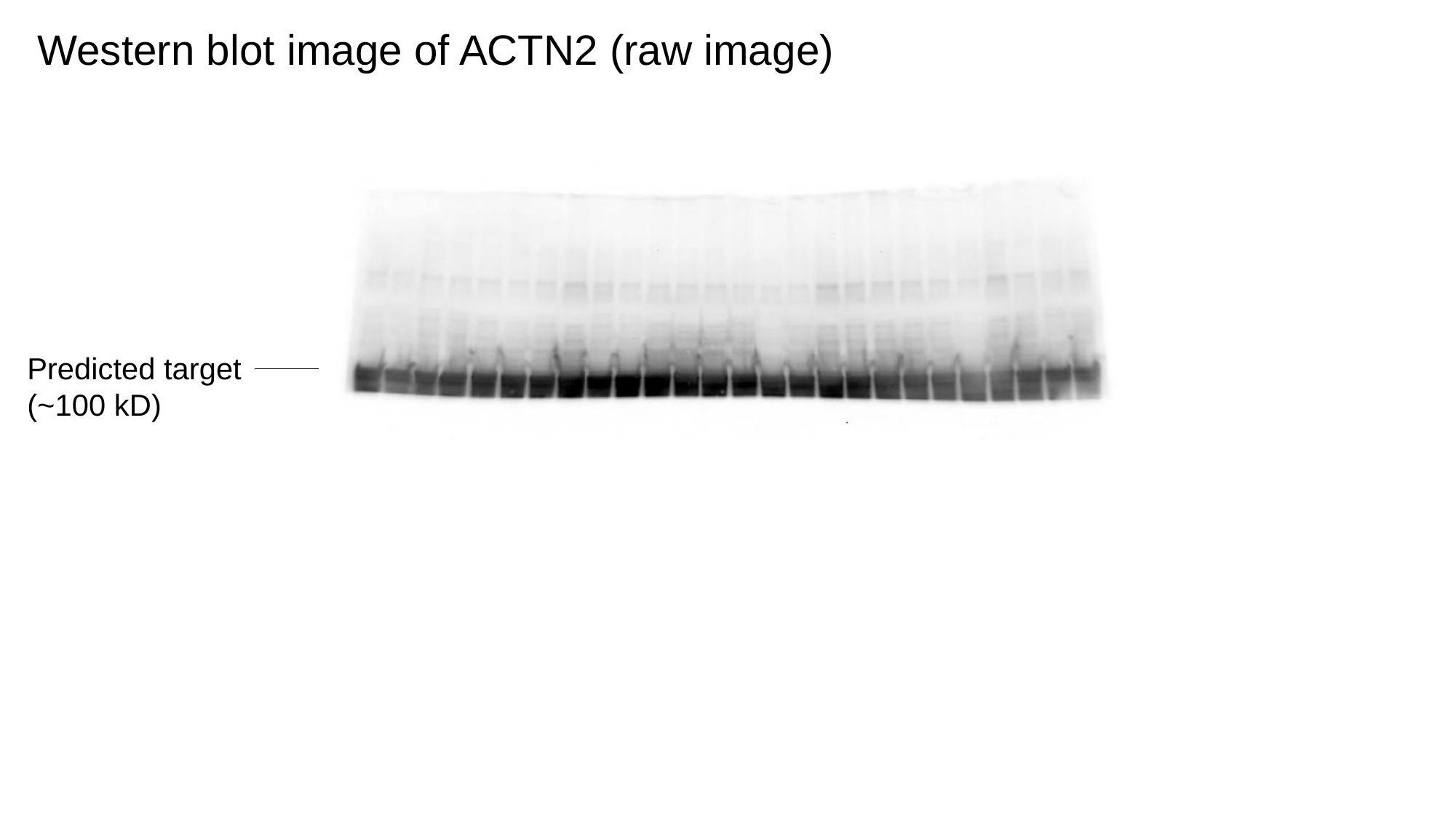

Western blot image of ACTN2 (raw image)
Predicted target
(~100 kD)

## Slide 3
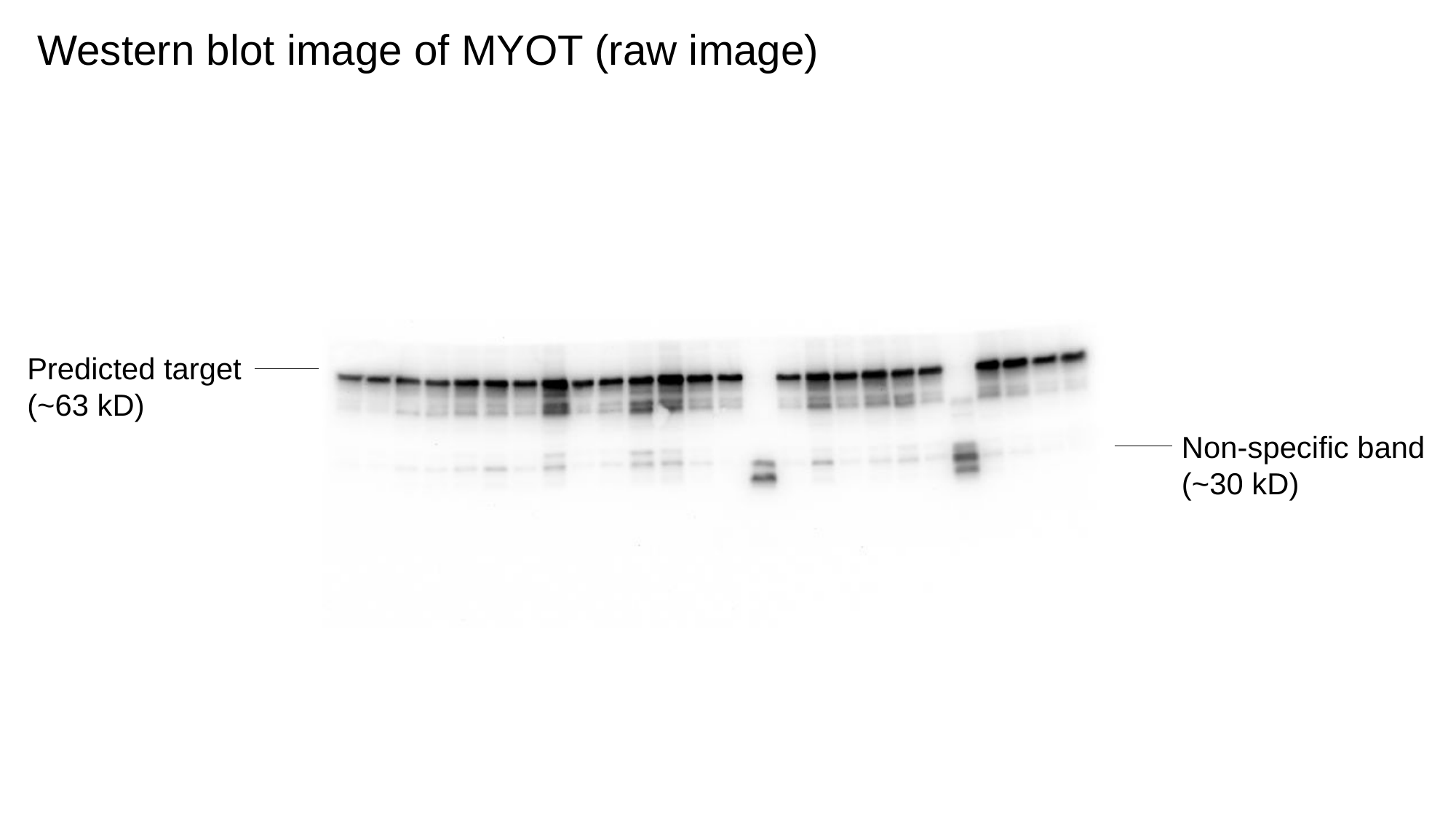

Western blot image of MYOT (raw image)
Predicted target
(~63 kD)
Non-specific band
(~30 kD)

## Slide 4
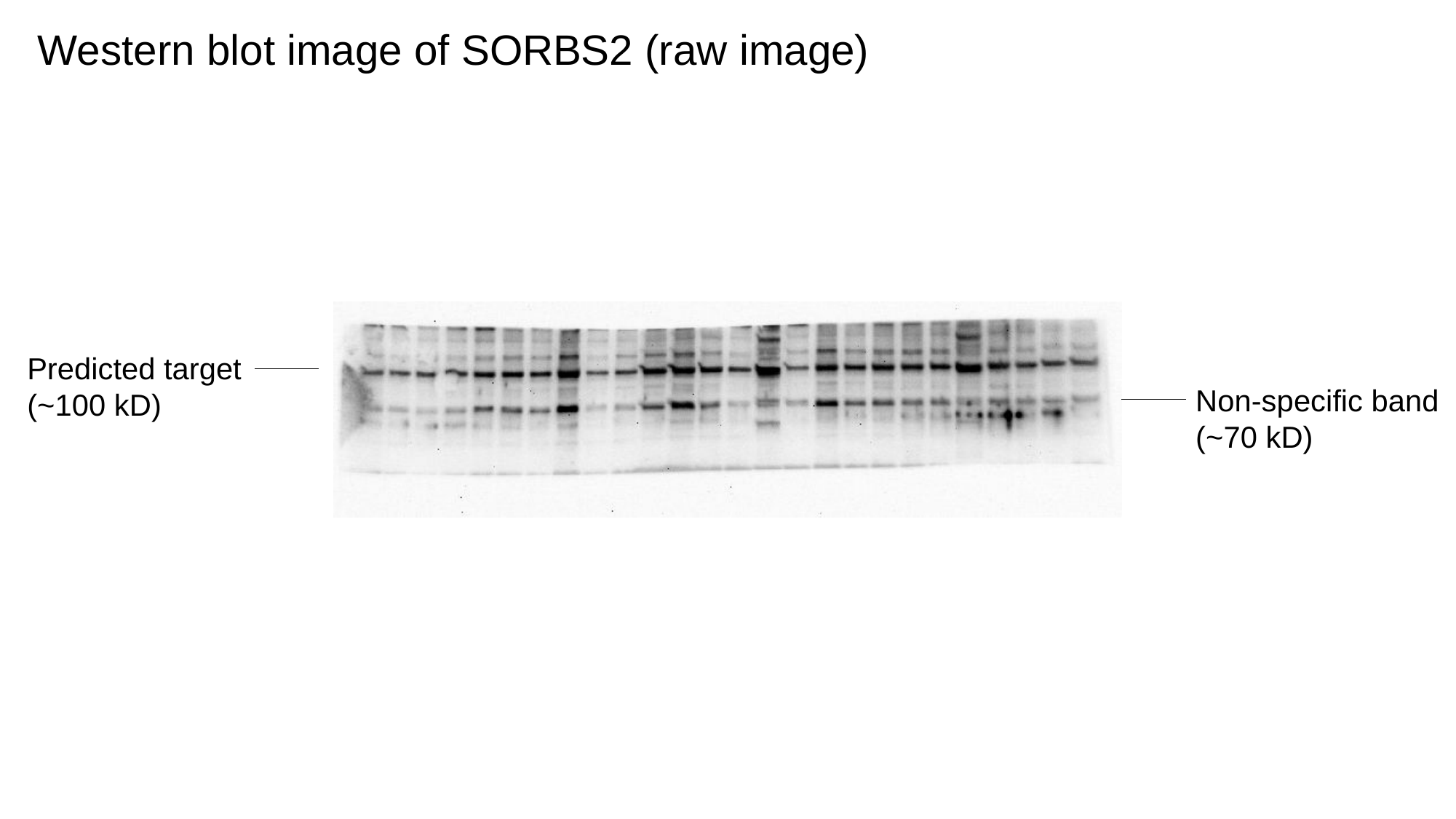

Western blot image of SORBS2 (raw image)
Predicted target
(~100 kD)
Non-specific band
(~70 kD)

## Slide 5
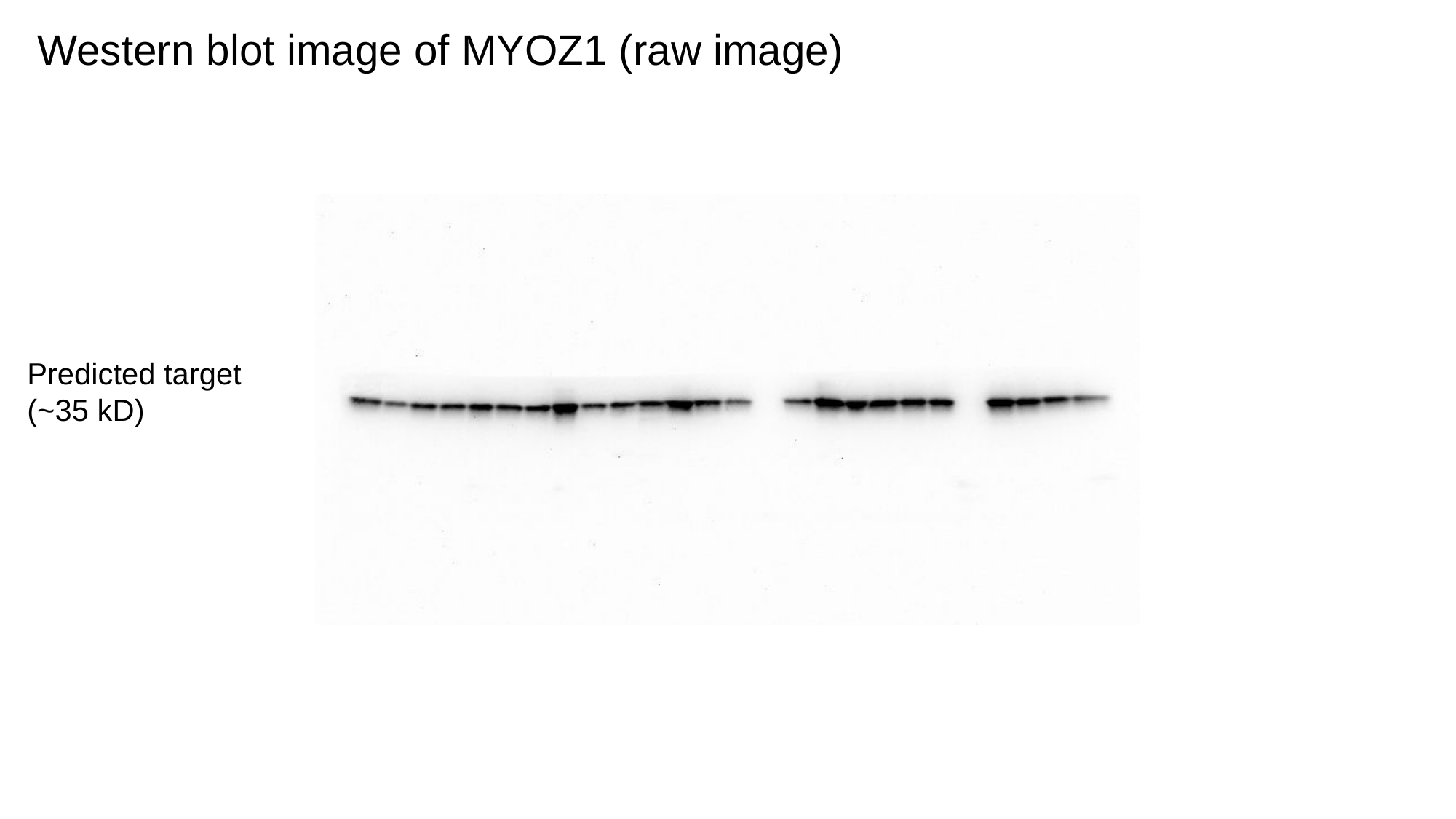

Western blot image of MYOZ1 (raw image)
Predicted target
(~35 kD)

## Slide 6
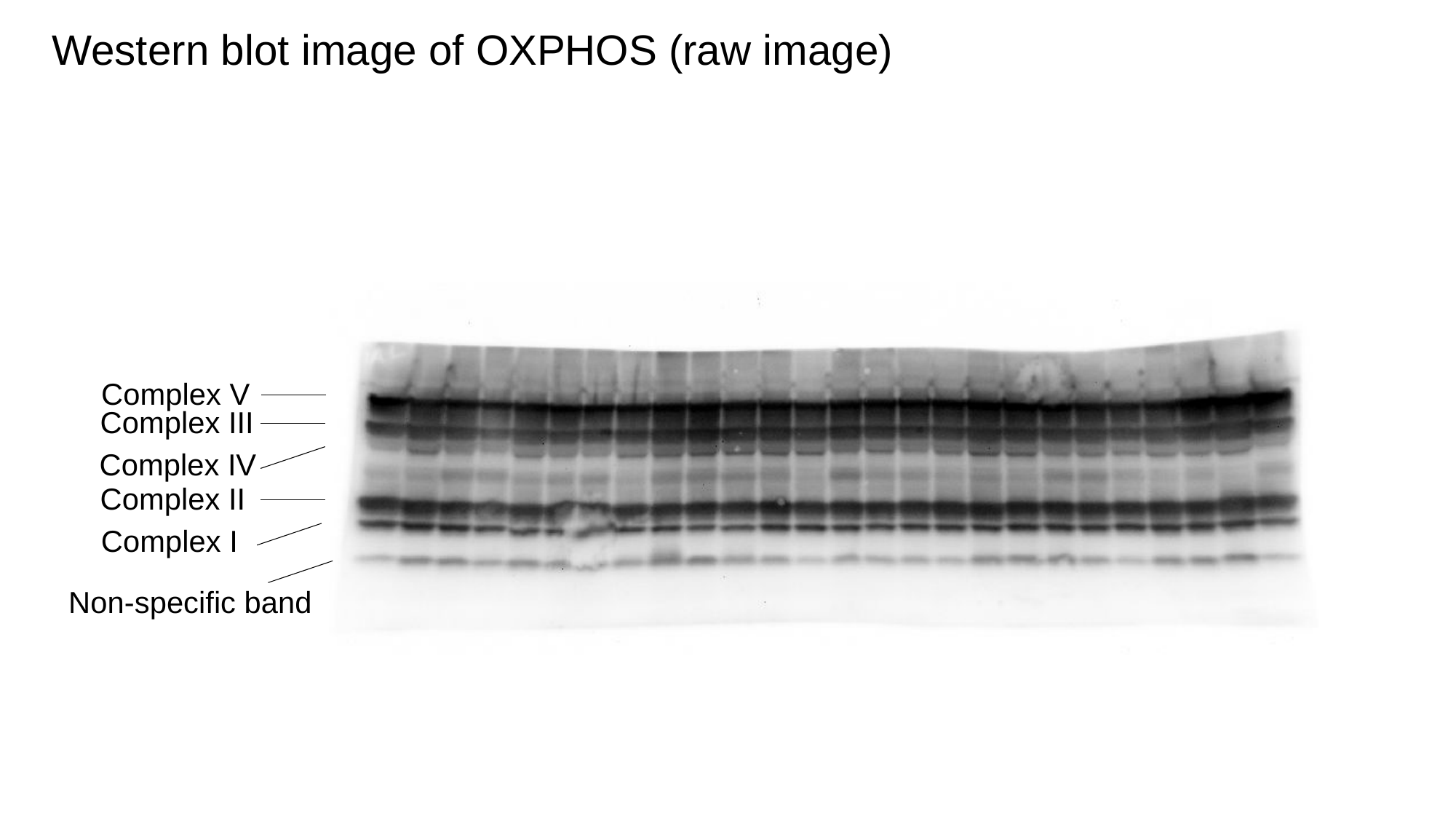

Western blot image of OXPHOS (raw image)
Complex V
Complex III
Complex IV
Complex II
Complex I
Non-specific band
